# Laparoscopic Gastric Sleeve in a Complex Elderly Patient: A Case Report

**DOI:** 10.7759/cureus.75690

**Published:** 2024-12-14

**Authors:** Christian Rodríguez-López, Sandy Martinez Castro, Jorge Vera Macías, Esperanza Escobosa-Rocha, Milton Alberto Muñoz Leija

**Affiliations:** 1 Bariatric Surgery, Body Art Bariatrics, Tijuana, MEX; 2 Bariatric Surgery, Hospital de Especialidades, Portoviejo, ECU; 3 Anesthesiology, Body Art Bariatrics, Tijuana, MEX; 4 General Surgery, Universidad de Monterrey, San Pedro Garza García, MEX; 5 Surgery, Body Art Bariatrics, Tijuana, MEX

**Keywords:** diabetes and bariatric surgery, diabetes mellitus in elderly, effect of bariatric surgery, elderly population, laparoscopic gastric sleeve gastrectomy

## Abstract

Obesity has been regarded as an epidemic in recent years. Various treatments have been developed, with bariatric surgery showing the highest levels of safety and effectiveness. This has increased its popularity and demand not only among young adults but also among elderly patients. Advances in medicine have extended the average lifespan, prompting patients over 65 years of age to seek bariatric procedures to enhance their quality of life and reduce comorbidities. Studies have demonstrated safety and effectiveness in this patient group; however, evidence is still limited for complex cases, particularly for those over 75 years old with multiple comorbidities. This study aims to describe a successful case of a geriatric patient with extreme obesity and multiple comorbidities who experienced an improvement in quality of life following a laparoscopic sleeve gastrectomy procedure in northwest Mexico.

## Introduction

Obesity represents a significant global health concern in contemporary society. In 2016, over 1.9 billion adults aged 18 and older were overweight, with over 650 million classified as obese [1]. The prevalence of obesity among adults aged 60 and above in Mexico stands at 32.2%. This surge, especially notable in older populations, poses critical implications for future public health policies [2]. Bariatric surgery has emerged as a pivotal intervention, demonstrating remarkable effectiveness in addressing this complex condition. It has significantly enhanced patients' quality of life and facilitated the complete remission or substantial improvement of obesity-related comorbidities. However, the efficacy and safety of bariatric procedures in individuals with multiple comorbid conditions, such as hypertension, diabetes, or heart disease, remain subjects of ongoing deliberation [3].

Within this context, we present a compelling case of an individual categorized as high-risk, although the definition of risk factors in bariatric surgery varies. High-risk patients, as outlined by Moulla et al. [4], include those with advanced age, extreme BMI, heart failure with a low ejection fraction (EF), liver cirrhosis graded Child A or B, end-stage renal failure requiring dialysis, or post-organ transplantation status. Utilizing the Metabolic and Bariatric Surgery Accreditation and Quality Improvement Program (MBSAQIP) [5], an online tool, the patient's overall risk for any complication was calculated at 11.40%, with a 7.41% probability of encountering a serious complication. In this case, our patient underwent laparoscopic sleeve gastrectomy (LSG), which is considered safe and effective for the treatment of obesity [6]. The decision to opt for LSG was based on a comprehensive evaluation of the patient's condition, considering its safety profile and effectiveness in similar high-risk cases. This case explores the challenges and outcomes of bariatric surgery in a patient with a complex medical history who underwent LSG. This study complies with the Surgical CAse REport (SCARE) guidelines [7].

## Case presentation

A 77-year-old male with a BMI of 41.31 kg/m^2^ and a weight of 130.9 kg presented with a complex medical history, including alcohol and sporadic caffeine use. He experienced multiple high-risk surgical events, including diastolic heart failure leading to an acute cardiac stroke, which resulted in coronary artery disease necessitating stent placement in 2007. Between 2014 and 2021, he underwent knee joint replacement surgeries. In 2018, open-heart surgery was performed due to pulmonary emboli. His clinical conditions included diabetes mellitus type 2, chronic kidney disease stage III, systemic arterial hypertension, dyslipidemia, diastolic heart failure grade III, sleep apnea, deep venous insufficiency, hypercoagulation, secondary hyperaldosteronism, pulmonary hypertension due to emboli, polyneuropathy, osteoarthrosis, gonarthrosis, and sensorineural hearing loss. His medication regimen was extensive, including insulin, amlodipine, carvedilol, furosemide, gabapentin, warfarin, loperamide, losartan, metformin, omeprazole, pravastatin, spironolactone, tadalafil, and testosterone.

Following comprehensive evaluations by various specialties, it was concluded that, given the high-risk nature of his comorbidities, he was a suitable candidate for LSG. This decision was informed by evidence suggesting bariatric procedures could mitigate complications associated with his existing conditions.

Preoperative laboratory studies did not demonstrate any eventuality. The patient had mild anemia (hemoglobin level of 12.3 mg/dL) and chronic kidney disease in category G3b (Chronic Kidney Disease Epidemiology Collaboration (CKD-EPI) glomerular filtration rate index of 38.05 ml/min/1.73m^2^).

**Figure 1:**
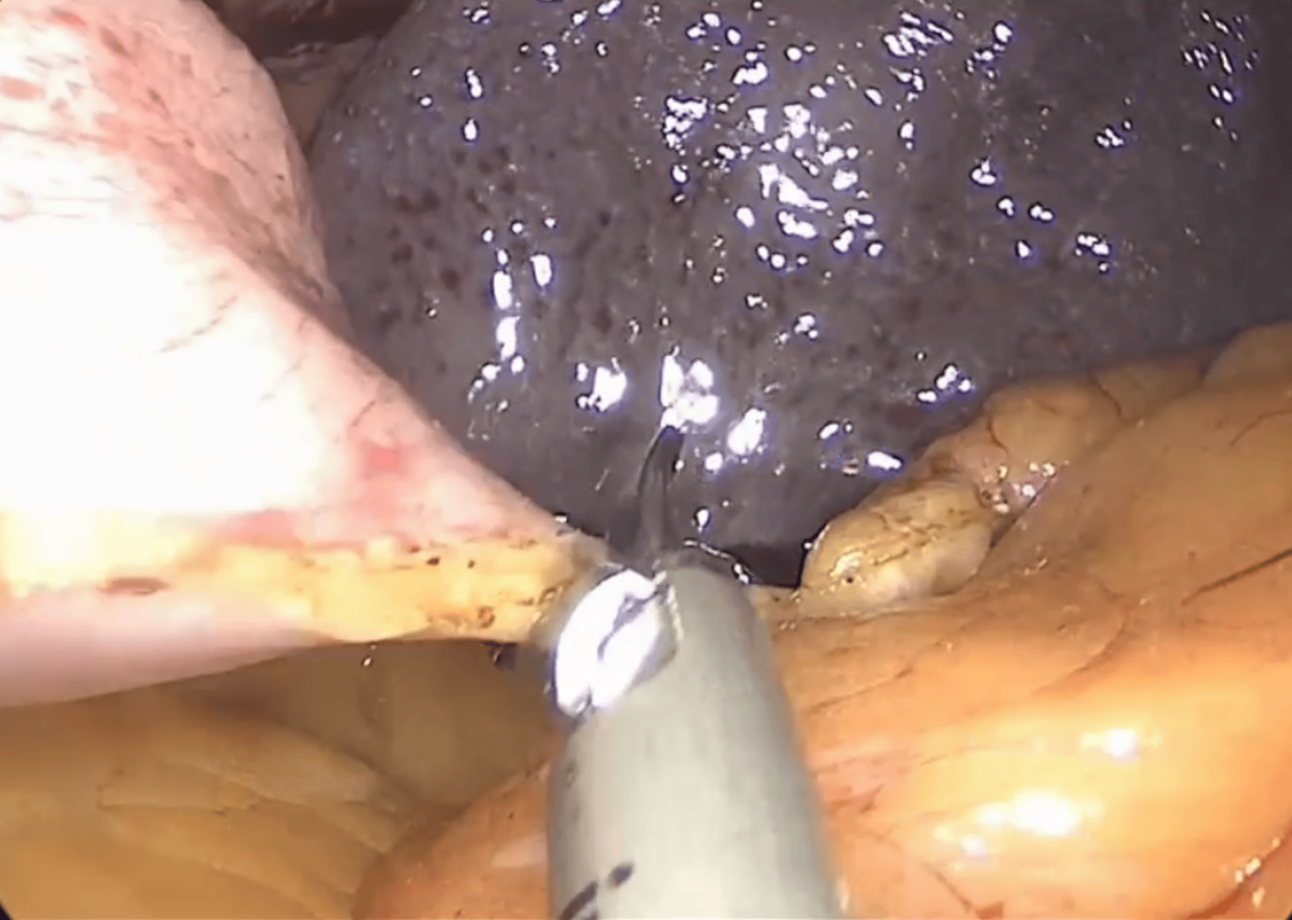
Resection of the greater omentum along the greater curvature of the stomach up to the gastric fundus area.

The LSG was performed at a high-volume hospital specializing in bariatric surgery. The procedure was performed under general anesthesia. The patient was positioned in the supine split-leg position (French position). The surgeons were positioned as follows: the primary surgeon in front of the patient (between the legs), the first assistant on the left side, and the second assistant on the right side. Using a supraumbilical Veress technique, pneumoperitoneum was created at 15 mmHg. A 10 mm trocar was placed at the same site. A diagnostic laparoscopy was performed, followed by the placement of 5 mm trocars (one on the left and one on the right, using the mid-axillary line as a reference point). A 12 mm trocar was placed at the left mid-clavicular line, and finally, a 5 mm trocar was placed subxiphoidally. Using a reference point approximately 4 mm from the pylorus, the greater curvature was released using ultrasonic energy. The greater omentum was mobilized up to the gastric fundus area (Figure 1).

**Figure 2:**
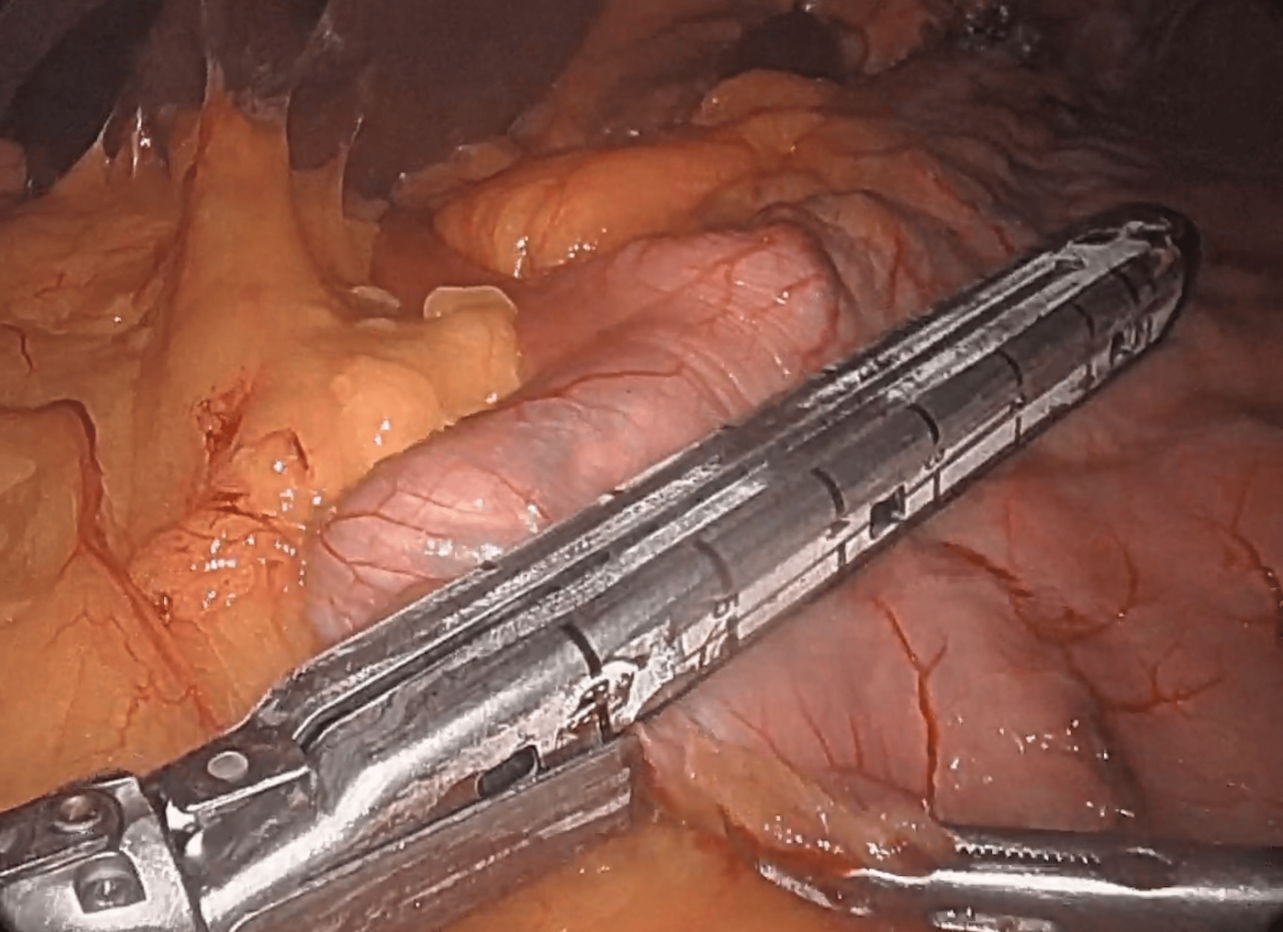
Use of a surgical stapler for the creation of the new stomach.

The new stomach was calibrated with a 32-Fr orogastric tube until the resection of the greater omentum began. Four surgical staplers (Medtronic, Dublin, Ireland) were used to create the new stomach (Figure 2).

Hemostasis was verified, and the staple line was reinforced with continuous invaginated sutures using 2-0 polypropylene. A closed drain was placed and externalized through the left 5 mm trocar.

**Figure 3:**
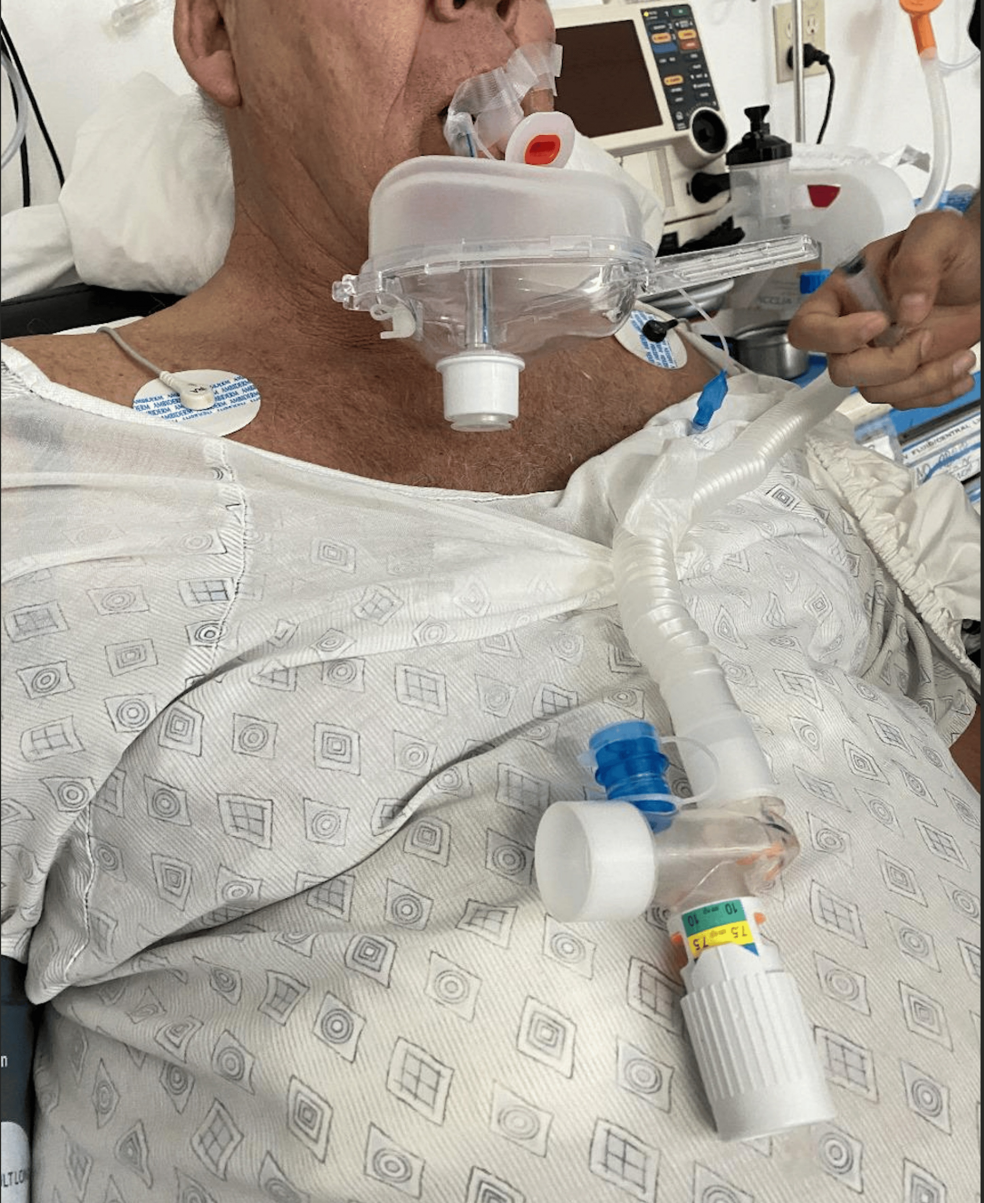
Use of CPAP during postoperative anesthetic care. CPAP: continuous positive airway pressure.

Anesthesia management was critical, with minimal doses of muscle relaxants administered. Pre-oxygenation for five minutes preceded sedation with a 30 mg bolus of propofol and an initial dose of 50 mcg fentanyl. Anesthetic induction included intravenous fentanyl 150 mcg, vecuronium 6 mg, and propofol 100 mg. Maintenance comprised sevoflurane 2% with 250 ml of trans-surgical saline solution containing buprenorphine 150 mcg, metamizole 1 gr, magnesium sulfate 2 gr, dexamethasone 8 mg, lidocaine 120 mg, ondansetron 8 mg, dexmedetomidine 50 mcg, and hyoscine 20 mg. Extubation was performed with the patient at a Ramsay scale of 2, reversing muscle relaxation with 1 mg of neostigmine and 1 mg of Atropine. The patient was then transferred to the post-anesthesia care unit and placed on continuous positive airway pressure (CPAP). Postoperative care included CPAP use and vigilant monitoring of vital signs (Figure 3).

The patient received 1000 milliliters of Hartmann's solution over 24 hours, and intravenous ceftriaxone 1 g was administered every 12 hours. Intravenous omeprazole 40 mg was given every 12 hours, complemented by intravenous ondansetron 8 mg every eight hours. Additionally, dipyrone 2 g plus metoclopramide 10 mg in 500ml of Hartmann's solution was administered intravenously over 30 minutes, followed by intravenous dipyrone 1 g every eight hours post-Hartmann's solution, and subcutaneous enoxaparin 60 mg every 12 hours post-drainage evaluation and quantification.

At the 24-hour postoperative assessment, the patient exhibited favorable progress. He reported mild pain at the surgical site and mild nausea, both effectively managed with medications. Early ambulation was initiated. With the serous drainage content, it was removed, and the patient was discharged at 48 hours without any complications. After 18 months of follow-up, the patient lost 32.5% of excess weight loss (EWL), and anti-hypertensive use reduced and removed insulin requirements.

## Discussion

This case report highlights the complexities and considerations in performing bariatric surgery on elderly patients with comorbidities. The outcomes of bariatric surgery in older adults, especially those over 65 years, have been a subject of interest in recent research. A study by Iranmanesh et al. (2022) found that postoperative complications in patients aged 65 and above were like those in younger patients, indicating the safety and efficacy of these procedures in the elderly [8]. Furthermore, the study reported significant improvements in comorbidities and weight loss at both one- and three-year follow-ups, despite the elderly having less pronounced improvements compared to younger cohorts.

Moreover, Vinan-Vega et al. (2018) conducted a retrospective case-control study at Mayo Clinic, which included 150 bariatric patients and found no significant difference in length of stay, short-term weight loss, and complication rates between patients aged 65 and older compared to younger patients [9]. This suggests that age alone should not be a deterrent in considering bariatric surgery for elderly patients.

However, it's important to consider that elderly patients might present with a different risk profile. Mabeza et al. showed that patients older than 65 years could experience higher mortality, morbidity, and resource consumption following bariatric surgery compared to younger adults [10]. This underlines the importance of a comprehensive preoperative evaluation and tailored perioperative management in this patient group.

Recent advancements in surgical techniques, such as robotic-assisted bariatric surgery, have shown promising results. A study evaluating the safety of robotic-assisted bariatric surgery in older adults using the MBSAQIP Database found that robotic sleeve gastrectomy (R-SG) was associated with lower odds of developing serious complications compared to other procedures [11]. This innovation could be particularly beneficial in elderly patients who are at higher risk of complications.

This case demonstrates the effectiveness of LSG in elderly patients. The efficacy of bariatric surgery has been established in patients over 65. However, very few studies evaluate complex or older patients like ours. A recent study includes a 78-year-old patient in its sample [12], while another mentions a 75-year-old patient [13]. However, these patients are not described in detail.

The complexity of these cases presents a significant challenge for bariatric surgeons, highlighting the importance of a specialized multidisciplinary team for this patient population. With the growth of medical tourism in our region and increasing life expectancy [14,15], it is crucial for bariatric surgeons to understand these complex cases and have treatment options available for such patients.

Although SG is a restrictive procedure, micronutrient deficiencies have been reported in the literature following the surgery. Deficiencies in iron, ferritin, folic acid, vitamin B12, magnesium, calcium, zinc, and phosphorus have been observed, highlighting the importance of considering supplementation for these patients both as a preventive measure and after the procedure [16,17].

One advantage of our study is that it is one of the few case reports to provide a detailed account of a patient's complex situation. It is also the first to describe a successful outcome in a patient over 75 years old in our country.

## Conclusions

This clinical case exemplifies the complexities and challenges associated with bariatric surgery in high-risk geriatric patients, particularly those with multiple comorbidities. This case reinforces current literature evidence that bariatric surgery can be a safe and effective option to enhance life quality in the geriatric population, even in the presence of challenging health conditions.

The gastric sleeve procedure appears to be a good option for complex patients with multiple comorbidities. With increasing life expectancy and the rise in medical tourism, it is important for bariatric surgeons to recognize that age and patient complexity are not contraindications for surgery. Appropriate facilities and a well-trained multidisciplinary team are essential for safely managing these types of patients.
